# Influence of passive, diet-based, hydration on muscle quantity assessment

**DOI:** 10.3389/fphys.2025.1724047

**Published:** 2025-11-21

**Authors:** Julian Seiler Viken, Vebjørn Nettum, Gøran Paulsen, Olivier Seynnes

**Affiliations:** 1 Department of Physical Performance, Norwegian School of Sport Sciences, Oslo, Norway; 2 Norwegian Olympic Federation, Oslo, Norway

**Keywords:** water content, muscle mass, muscle volume, swelling, ultrasound, DXA

## Abstract

**Background:**

This study investigated the impact of passive changes in hydration level on measurements of lean tissue mass (LTM) and muscle morphology.

**Methods:**

Sixteen healthy participants (13 males, 3 females) completed two testing sessions 24 h apart, including dual-energy X-ray absorptiometry (DXA), 3D ultrasound imaging of the vastus lateralis (VL), and bioelectrical impedance analysis (BIA). Participants fasted for 12 h before each session. Hydration was manipulated by assigning participants randomly to either a non-hydrated state, abstaining from fluids until testing, or a hydrated state, consuming water equal to 1.5% of body mass 2 hours prior.

**Results:**

Total body water, measured using bioelectrical impedance analysis (BIA), was lower in the non-hydrated condition (48.6 ± 9.7 kg) than in the hydrated condition (49.0 ± 9.8 kg, p = 0.015). In the hydrated condition, total LTM, measured using DXA, was higher for the full body (60.8 ± 11.8 kg vs. 60.0 ± 11.9 kg, p < 0.001) and the trunk (29.2 ± 5.3 kg vs. 28.6 ± 5.4 kg, p < 0.001). Hydration did not significantly affect LTM in the upper (p = 0.251) or lower limbs (p = 0.298), or the thigh (p = 0.051). Volume (p = 0.105) and cross-sectional area (CSA; p = 0.114) of the VL were also unaffected.

**Conclusion:**

Small changes in hydration levels induced passively under temperate conditions may not significantly affect measurements of muscle morphology or limb measurements of LTM. However, monitoring changes in muscle quantity with total LTM may be biased by water intake and trunk hydration if these factors are not controlled. Caution is warranted (e.g., standardisation) when using total LTM to monitor changes in muscle quantity.

## 1 Introduction

Accurate assessment of changes in contractile material is important when assessing various clinical conditions (e.g., diseases causing muscle wasting) and in research applications (e.g., evaluating the effectiveness of interventions). Such assessments can be done non-invasively with different techniques, based on measurements of lean mass content or muscle size. Dual-energy X-ray absorptiometry (DXA) is a widely used method for assessing lean tissue mass (LTM) (Meyer et al., 2013), while muscle morphology is typically assessed with magnetic resonance imaging or, since recently, with 3D ultrasonography (Huet et al., 2024). Despite their broad intrinsic differences, each method is considered valid and reliable, and based on purposes and availability, they are often used together or interchangeably (Slater et al., 2022; Frouin et al., 2023; Herberts et al., 2023). However, their comparative susceptibility to bias under certain conditions, such as hydration levels and fluid shifts, is less documented. Hydration levels can fluctuate due to various factors, including prolonged exercise (Sawka et al., 2007), submaximal exercise (Sjøgaa et al., 1985), dietary intake (Shiose et al., 2022; Bone et al., 2017), and pathology (Cheng et al., 2023). Such changes in hydration status may influence both intramuscular fluid volume and muscle size measurements, by altering osmotic pressure and/or increasing hydrostatic force (Appell et al., 2025). While factors contributing to dehydration (environment, exercise) are usually controlled, the impact of passive variations in hydration levels (daily fluctuations in fluid intake) on muscle quantity measurements is often not considered.

Direct evidence regarding the impact of passive dehydration/hydration on muscle morphology is lacking. However, body composition measurements like DXA, have been shown to be sensitive to changes in total body water content during passive dehydration/rehydration (Going et al., 1993). For instance, passive dehydration can induce an approximately 2% decrease in body weight, leading to a significant reduction in LTM, which returns to baseline after rehydration. Importantly, DXA does not directly measure muscle dimensions. Instead, it relies on prediction equations calibrated under the assumption of stable hydration levels (Rodriguez et al., 2024). These findings emphasize that even small, daily fluctuations in hydration status can affect measurements involving soft, water-rich tissues, although this effect is not characterized for local measurements.

Many research studies and clinical assessments rely on LTM and muscle morphology measurements, where hydration status could act as a confounding factor, potentially affecting the interpretation of results. A comparative study assessing the effect of dietary-induced variations in hydration levels on LTM and muscle volume could inform methodological choices. This study aimed to compare the impact of hydration status on LTM and muscle volume measurements using DXA and 3D ultrasonography in healthy, recreational to trained individuals. We hypothesized that the small change in intramuscular water content caused by passive dehydration/rehydration would affect LTM measurements from DXA but would not appreciably influence measurements of muscle morphology.

## 2 Materials and methods

### 2.1 Ethics approval

All participants received comprehensive information about the research project and gave written informed consent prior to participation. The study was approved by the ethical committee of the Norwegian School of Sport Sciences (approval number: 362 - 061124).

### 2.2 Participants and experimental design

Sample size calculations indicate that a minimum detectable change (MDC) of 3.4 mL for 3D ultrasound measurements of the vastus lateralis (VL) muscle volume (Ritsche et al., 2025) requires a sample size of n = 10 for a two-tailed paired t-test, with an alpha level of 0.05 and a power of 0.8. To account for possible dropouts, sixteen trained adults (13 males, 3 females; height: 179 ± 11 cm; body mass: 77 ± 14 kg; age: 25 ± 3 years) participated in this study and were classified as Tier 1 (recreationally active individuals) or 2 (trained or developmental athletes) according to McKay et al. (2022) (McKay et al., 2022). Exclusion criteria consisted in musculoskeletal disorders or occurrences of injuries of the thigh muscles over the 6 months prior to testing. The experimental protocol consisted in testing the variability of measurements of muscle morphology and LTM under contrasting conditions of hydration. A passive model of (de)hydration was adopted to reflect expectable fluctuations due to dietary habits. Water intake was manipulated, which we assume could influence hydration status. Participants underwent three scanning sessions within 1 week, each preceded by a 12-h fast. All assessments were performed in the morning between 8 and 10 a.m. Participants were instructed to maintain their usual dietary regimen in the 24–48 h preceding each testing session and to avoid any major changes in food or fluid intake. Sodium intake and glycogen status were not standardized across participants. The first two sessions were conducted 24-h apart, with the order randomly assigned. Each session included a DXA scan, 3D ultrasound imaging of the VL, and bioelectrical impedance analysis (BIA). On the morning of the hydrated condition day, participants ingested a volume of water equivalent to 1.5% of their body mass (e.g., 1.2 L for an 80 kg individual) 2 hours prior to testing, while on the non-hydrated condition day, they refrained from fluid intake. A change of ∼1.5% in body mass is below the typical ≥2% threshold used to define hypohydration, yet above normal daily variation in body water (∼≤1%) and is therefore considered a small but meaningful change in hydration status (Cheuvront and Kenefick, 2014; Greenleaf, 1992). The third session, 24-to-48 h after the second one, was dedicated to collecting reliability data for the 3D ultrasound technique, which was newly implemented in the lab, and was conducted under the non-hydrated condition for standardized comparison. Each test day began with height and body mass measurements using an electronic scale and fixed stadiometer (Seca, Hamburg, Germany). Participants were instructed to avoid exercise for 24 h before each test day to maintain consistency in test conditions.

### 2.3 Muscle volume and CSA

#### 2.3.1 3D ultrasound imaging setup

Two-dimensional ultrasound images were acquired using a brightness mode ultrasonography device with a 5-18MhZ transducer (50 mm, Mach 30, Hologic SuperSonic Imagine, Aix-en-Provence, France). The imaging depth was set to 7 cm and presets including 49% gain, 65 dB dynamic range, persistence off, and no external probe pressure. Real-time video capture of ultrasound images was conducted using a video grabber (ElGato Cam Link, Corsair Components, Fremont, CA, USA). A 3D-printed stand-off with 4 reflective markers was attached to the transducer. The spatial position and orientation of the transducer were tracked using an optoelectronic motion capture system (<100 Hz>, 8 cameras, Optitrack Flex 3, NaturalPoint, Corvallis, OR, USA). Synchronization between the ultrasound and motion capture data was achieved using the open-source PlusServer software (Public Software Library for US Imaging Research, version 2.8.0, Kingston, ON, Canada). Data acquisitions were recorded using open-source software 3D Slicer (slicer.org, version 5.6.2, Perth, Australia). Temporal calibration was performed using open-source software fCal (Freehand Tracked US Calibration Application, version 2.9.0, Kingston, ON, Canada), while spatial calibration was done with the 3D Slicer software (Frouin et al., 2023).

#### 2.3.2 Muscle volume acquisition

**FIGURE 1:**
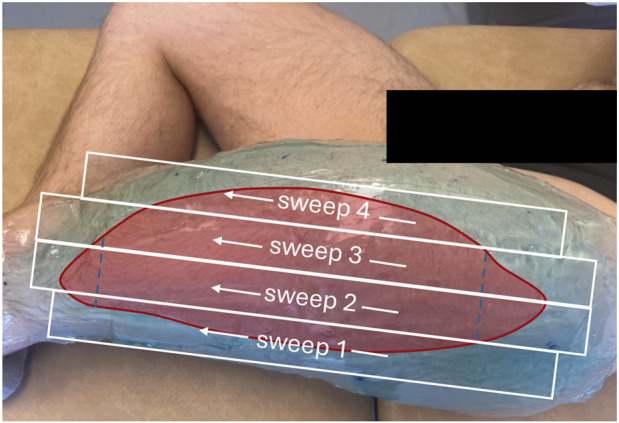
Participant positioned for ultrasound scanning with a coat of gel of about 1 cm. The red overlay represents the area of the vastus lateralis muscle. The white boxes denote the probe sweep trajectories and the dashed lines indicate the distal ends of the rectus femoris and tensor fascia latae, respectively.

The left VL was imaged with participants positioned horizontally on one side, lying sideways to minimize movement and optimize scanning coverage, as determined during pilot testing. Prior to data acquisition, the VL origin, insertion, and medial and lateral boundaries were identified and marked using a marker. To minimize tissue compression and enhance image quality, a 1-cm coat of ultrasound gel was applied evenly with a plastic scraper (Figure 1). Four to six parallel scanning sweeps were performed at a constant speed, following a standardized scanning technique. All scans were performed by the same experienced examiner (JSV), who completed approximately 100 h of training prior to data collection.

#### 2.3.3 Muscle cross-sectional area segmentation and volume reconstruction

**FIGURE 2:**
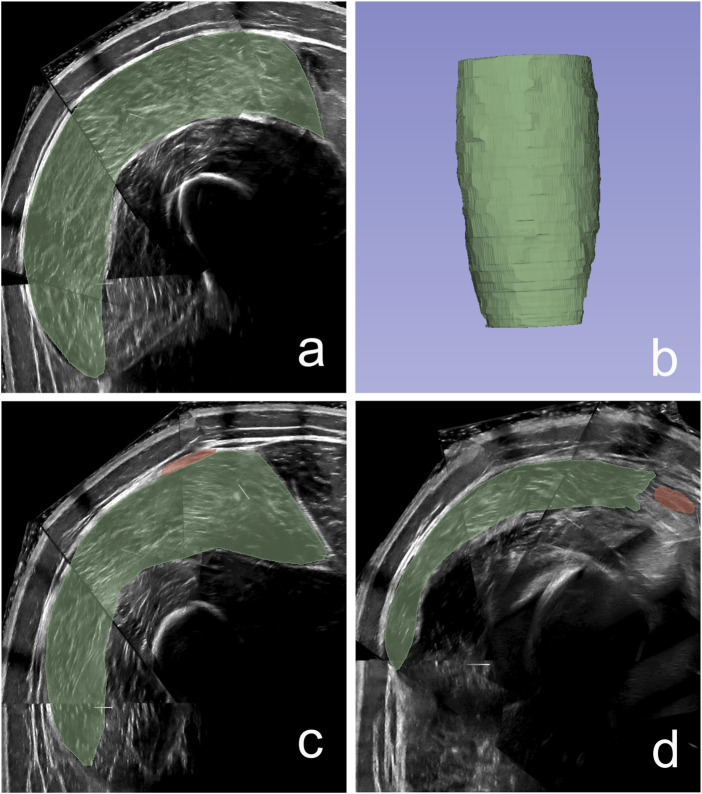
**(a)** Example of a cross-sectional area slice of the segmented vastus lateralis, **(b)** 3D visualization of the reconstructed muscle volume, **(c)** First segmentation slice at the insertion of the tensor fascia latae, **(d)** Final segmentation slice at the insertion of the rectus femoris.

Ultrasound image reconstruction was performed using the *Volume Reconstruction* module in 3D Slicer (Fedorov et al., 2012). Voxel sizes were set at 0.20 × 0.20 mm for the transverse plane and 1.0 mm for the longitudinal axis. The area of the VL was manually delineated along the muscle length at average intervals of approximately 7 mm. Additional regions of interest were generated in the software, with the interpolation mode set to *Linear*, the optimization mode set to *Full optimization*, the compounding mode set to *Mean*, and the *Fill holes* option was checked. Pilot testing indicated that the volume reconstruction did not render the proximal and distal ends of the muscle as accurately as the rest of the muscle belly. To increase reliability, the volume was calculated between the distal end of the rectus femoris and the tensor fascia latae muscles (Figure 2). Cross-sectional area (CSA) measurements were obtained from the muscle volume, using the *Segment Cross-Section Area* module from the 3D Slicer *Sandbox* extension package. The position of the largest CSA relative to the analyzed proximo-distal boundaries of the muscle in the non-hydrated condition was first identified. The corresponding CSA in the hydrated condition was then identified as the CSA at the same relative position. Segmentations were performed by a single experimenter (JSV), who was blinded to both the participant and the experimental condition.

### 2.4 Dual-energy X-ray absorptiometry

Lean tissue mass was assessed using a DXA scanner in full body mode (Lunar iDXA (GE Healthcare) and the GE Encore software (version 13.60, GE Healthcare). The standard thickness mode was utilized for all scans. Daily calibrations were conducted based on the manufacturer’s guidelines before measurements. Participants were positioned in a supine position with hands semi-pronated, and with a standardized gap distance between the arms and the trunk and between their legs. The position of the lower limbs was standardized by fastening the knees and ankles. All participants wore minimal clothing for accurate assessment. Standard segmentation of the arms and legs was adjusted individually based on the shoulder and hip joints, respectively. In addition, a custom segmentation of the left thigh was performed, with the segment defined by the proximo-distal boundaries of the femur.

DXA is considered a reliable method for assessing body composition, demonstrating an inter-session coefficient of variation (CV) of 0.5% and a least significant change (LSC) of 0.7 kg for whole-body lean mass (Slater et al., 2022).

### 2.5 Bioimpedance impedance analysis

Total body water was assessed from electrical impedance measurements (In-body 720, Biospace Co. Ltd., Seoul, Republic of Korea). Measurements were taken in accordance with the manufacturer’s calibration guidelines. Participants stood with their feet on the platform electrodes. Their arms were extended at a 15° angle to each side while gripping the handles, ensuring contact with the electrodes. Electrical currents were applied at frequencies of 1, 5, 50, 250, 500, 1000 kHz while the participants stood still to assess impedance. The inclusion of BIA was intended to provide a measure of variations in water balance that would be independent of DXA-based outcome variables. In line with this, BIA has been shown to be sensitive to small, passive reductions in body water; for example, a study in physically active men demonstrated that a 0.76 kg body mass loss due to 5 h of passive dehydration slightly decreased fat mass and increased extracellular water and phase angle (Aburto-Corona et al., 2024). BIA is considered a reliable method for assessing body water content, demonstrating an inter-session CV of 0.6% and a LSC of 0.9 kg (Herberts et al., 2023).

### 2.6 Statistical analysis

Inter session reliability of VL muscle volume and CSA was assessed using the intraclass correlation coefficient (ICC) calculated as ICC (Slater et al., 2022; Meyer et al., 2013); a two-way mixed-effects model for absolute agreement of single measurements (Shrout and Fleiss, 1979) and coefficient of variation (CV). ICC values were interpreted according to Koo and Li (2016) as follow: low (≤0.50), moderate (0.50–0.75), (good ≥0.75), and excellent (≥0.90) (Koo and Li, 2016). The MDC was calculated as standard error of measurement (SEM) × √2 × 1.96. A Shapiro-Wilk test revealed that VL volume, total LTM and thigh LTM measurements were not normally distributed, whereas all other variables were. A non-parametric Wilcoxon test was used to compare non-normally distributed data between the hydrated and non-hydrated conditions, while paired-sample t-tests were used for all other comparisons. Effect size (ES) was measured as the Cohen`s d for the t-test and rank biserial-correlation for the Wilcoxon test. All results are presented as mean ± standard deviation (SD). The significance level was set at p < 0.05, and all statistical analyses were conducted using JASP (version 0.19.3, JASP, Amsterdam, Netherlands).

## 3 Results

**FIGURE 3:**
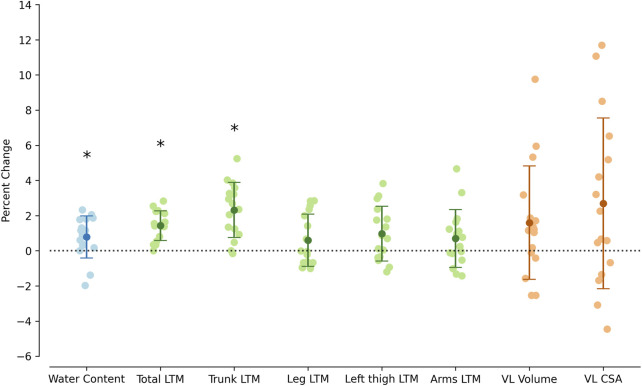
Percent differences between non-hydrated and hydrated conditions in total body water content, total lean tissue mass (LTM), trunk LTM, leg LTM, left thigh LTM, arms LTM, vastus lateralis (VL) volume, and VL cross sectional-area (CSA). Error bars represent the mean ± SD. *p < 0.05.

The mass of total body water as measured with BIA was 400 g greater in the hydrated compared to the non-hydrated condition (p = 0.015, Table 1; Figure 3). Body mass was also higher in the hydrated condition (p < 0.001). DXA measurements of total LTM and trunk LTM were higher in the hydrated condition (p < 0.001 in both cases, Table 1; Figure 3). However, no differences were found for LTM measurements in the arms (p = 0.251), leg (p = 0.298), or the thigh (p = 0.051).

**TABLE 1:** Comparison of body composition, muscle volume, and muscle cross-sectional area between hydrated and non-hydrated conditions.

Parameter	Method	Hydrated	Non-hydrated	ES	95% CI	p value
		Mean ± SD	Mean ± SD			
Body mass (kg)	Scale	78.0 ± 13.6	77.1 ± 13.6	2.0	[1.1, 2.8]	**<0.001**
Total LTM (kg)	DXA	60.8 ± 11.8	60.0 ± 11.9	1.8	[1.0, 2.6]	**<0.001**
LTM trunk (kg)	DXA	29.2 ± 5.3	28.6 ± 5.4	1.6	[0.8, 2.3]	**<0.001**
LTM leg (kg)	DXA	12.2 ± 2.5	12.1 ± 2.5	0.3	[-0.2, 0.7]	0.298
LTM thigh (kg)	DXA	9.0 ± 1.9	8.9 ± 1.9	0.6	[0.0, 0.8]	0.051
VL volume (mL)	3D-US	447 ± 124	440 ± 122	0.5	[-0.1, 0.8]	0.105
VL CSA (cm2)	3D-US	29.1 ± 8.1	28.5 ± 8.2	0.4	[-0.1, 0.9]	0.114
TBW (kg)	BIA	49.0 ± 9.8	48.6 ± 9.7	0.7	[0.1, 1.2]	**0.015**

The bold values represent significant effects.

Non-Hydrated refers to the condition without fluid intake; LTM, lean tissue mass; VL, vastus lateralis; CSA, cross sectional-area; TBW, total body water; DXA, Dual-Energy X-Ray Absorptiometry; 3D-US, 3D Ultrasound; BIA, bioelectrical impedance analysis; ES, effect size; CI, confidence interval.

Inter-session reliability for VL muscle volume and CSA is presented in Table 2. The ICC were excellent for both volume (0.997; 95% CI [0.991, 0.999]) and CSA (0.977; 95% CI [0.935, 0.992]), with a low CV for each measure (1.5% and 3.0%, respectively). The MDC was 19.1 mL for the muscle volume and 3.4 cm^2^ for CSA. Neither muscle volume (p = 0.105) nor muscle CSA (p = 0.114) changed between conditions (Table 1; Figure 3).

**TABLE 2:** Inter-session reliability of vastus lateralis muscle volume and cross-sectional using 3D ultrasound.

Muscle	Session 1	Session 2	CV (%)	ICC	MDC
VL volume (mL)	432.1 ± 134.8	427.5 ± 132.1	1.5	0.997	19.1
VL CSA (cm2)	27.5 ± 8.8	27.2 ± 7.7	3.0	0.977	3.4

VL, vastus lateralis; CSA, cross sectional-area; CV, coefficient of variation; ICC, intraclass correlation coefficient; MDC, minimum detectable change.

## 4 Discussion

The present results suggest that DXA measurements of LTM, including the trunk, were influenced by passive diet-based hydration, despite only minimal changes in body mass (∼1%) and body water content (∼0.5%). However, localized measurements of limb LTM or muscle size remained unaffected by these minor variations in hydration. These findings suggest that small variations in hydration may not appreciably bias muscle size and limb LTM measurements. They may, however, sway any LTM measurement, including the trunk segment.

While all outcome variables were lower in the non-hydrated condition, most differences did not reach statistical significance, indicating that the fluid shift did not substantially affect the muscular compartments. Previous research has shown that more extreme forms of dehydration or rehydration, particularly those involving heat exposure (Périard et al., 2021) or exercise (Ploutz-Snyder et al., 1995), can lead to significant changes in muscle size and lean body mass. In contrast, significant effects were observed in the trunk and total LTM in this study, whereas regional measurements, such as those in the legs and thighs, remained stable.

A potential overestimation of LTM by DXA may have occurred due to limitations in its calibration. Since the method does not differentiate between muscle mass and water, but instead provides LTM as a combined measure (Ellis, 2000), fluctuations in hydration status should be taken into account when interpreting DXA results. Both dehydrated or particularly well-hydrated measurement could lead to misinterpretation of changes, potentially masking true physiological adaptations or exaggerating them, underlining the importance of standardization protocols (Wang et al., 1999). These findings highlight the need for caution when interpreting DXA-derived measurements in conditions where hydration levels may vary, even minimally. However, this potential bias appears less likely to affect limb-level LTM measurements or muscle size assessments. In practice, this standardization should involve controlling the timing of both fluid and food intake, as well as avoiding strenuous exercise for at least 24 h prior to testing.

To reliably detect small differences in muscle volume and size between conditions, precise measurements are essential. The reliability of 3D ultrasound for the VL was excellent, with a CV of 1.5% for volume and 3.0% for CSA, intraclass correlation coefficients of 0.997 and 0.977, and MDC values of 19.1 mL and 3.4 cm^2^, respectively. The observed differences between conditions (−7 mL for volume and −0.6 cm^2^ for CSA) were well below these MDC thresholds, indicating that any hydration-related effects were not large enough to be detected. These reliability metrics are comparable to those reported for both 3D ultrasound and MRI, reinforcing the utility of 3D ultrasound as a reliable method for muscle size assessment under conditions where hydration status may vary (Huet et al., 2024). Additionally, the findings underscore the complexity of fluid shifts in the body and their impact on measurement accuracy. While no significant changes were observed in muscle volume or CSA, fluid shifts could still have a transient effect on muscle thickness, as shown by the 8% decrease in the vasti muscle group CSA after passive dehydration protocol combined with heat exposure (Reece et al., 2023). This decrease could be attributed to the combined effect of increased osmotic pressure and capillary hydrostatic pressure (Appell et al., 2025). In our study we only used a passive dehydration protocol, which mainly affects the capillary hydrostatic pressure, suggesting that minor changes in hydration may not impact muscle morphology directly, but could be influenced by the conditions under which measurements are taken.

Despite the absence of significant changes in muscle morphology, great inter-individual variability was observed (Figure 3). Although this variability was high, the bias introduced by dietary passive hydration is unlikely to have influenced the results. However, this assessment may need to be re-evaluated if greater variations in water content are expected, such as with certain types of exercise or prolonged heat exposure, or if morphological methods with a greater resolution are employed.

This study provides valuable insights into the sensitivity of muscle morphology measurements, such as muscle volume and CSA, to fluctuations in hydration within the expectable range of daily variations. Specifically, it investigates how small changes in water intake can impact assessments of muscle atrophy and hypertrophy. However, several limitations should be noted. One major limitation is that hydration itself was not directly measured (e.g., using urine, blood, or saliva), but instead, body mass and water content were used as proxies for hydration status. It is important to recognize that BIA, particularly in the standing position, does not ensure fully accurate fluid assessment, and TBW is not a direct marker of hydration (Armstrong et al., 2010; Blue et al., 2023). Therefore, while reductions in body mass and estimated water content suggest a relative decrease in total body water, this cannot be interpreted as verified dehydration. The data collected in this study can only be interpreted in the context of small hydration variations. The study did not test multiple levels of dehydration and rehydration, which limits the generalizability of the findings to more extreme fluctuations in hydration. While this design enhances ecological validity for typical daily variations, it should be interpreted with caution when applied to more extreme fluid shifts. Additionally, variability in hydration levels could have arisen due to differences in waking times and individual factors. Although participants were instructed to drink 1.5% of their body mass in liters upon waking, this standardization method was not perfect. To minimize potential bias, testing was conducted within a controlled time window (8–10 a.m.), though individual variability in hydration status at baseline may still have influenced the results. Furthermore, our 3D ultrasound assessments deliberately excluded the distal and proximal ends of the muscle to enhance measurement reliability. We recognize that if hydration changes in these regions differed, it could limit the comparability of our findings with other studies that included the entire muscle volume. Additionally, the study included a predominance of male participants, which could theoretically influence the results. Although some studies report sex-related differences in fluid loss following exercise (Eijsvogels et al., 2013; Wickham et al., 2021), short-term fluid redistribution to the muscle after water ingestion under resting conditions in males and females have not been investigated. To ensure that our findings were not influenced by the small number of female participants, we re-ran the main analyses excluding these participants, and the pattern of results remained unchanged.

## 5 Conclusion

Water intake significantly influenced certain LTM measurements obtained from DXA, while ultrasound-based measurements of muscle volume and CSA remained stable. These findings support the hypothesis that DXA is sensitive to hydration-related fluid shifts, while localized muscle morphology measurements may not be impacted by hydration status. In practical terms, our results suggest that standardizing hydration may not be as critical for accurate muscle morphology assessments in healthy, trained individuals. These findings have important implications for sport science research (e.g., athlete monitoring), applied sport practice (e.g., training evaluation), and clinical settings. In research studies, particularly those investigating muscle adaptations, controlling for hydration status is important, but it should not overshadow the need to account for more prominent factors such as exercise and heat exposure. In clinical settings, particularly for patients at risk of significant variations in hydration status (e.g., aging populations or individuals with conditions that affect fluid balance), caution should be exercised when using DXA for total LTM assessments. Instead, localized limb LTM or muscle morphology measurements may not be significantly affected by small changes in hydration levels induced passively under temperate conditions. This approach would provide a more accurate reflection of muscle changes, avoiding the potential bias introduced by hydration fluctuations.

## Data Availability

The raw data supporting the conclusions of this article will be made available by the authors, without undue reservation.
